# Testing delayed, gradual, and temporary treatment effects in randomized single-case experiments: A general response function framework

**DOI:** 10.3758/s13428-023-02230-1

**Published:** 2023-09-25

**Authors:** Rumen Manolov, Patrick Onghena

**Affiliations:** 1 Department of Social Psychology and Quantitative Psychology, Faculty of Psychology, University of Barcelona, Passeig de la Vall d’Hebron 171, 08035 Barcelona, Spain; 2 Faculty of Psychology and Educational Sciences, Methodology of Educational Sciences Research Group, KU Leuven, Tiensestraat 102, 3000, Leuven, Belgium

**Keywords:** Single-case experimental designs, Immediacy, Latency, Randomization

## Abstract

Randomization tests represent a class of significance tests to assess the statistical significance of treatment effects in randomized single-case experiments. Most applications of single-case randomization tests concern simple treatment effects: immediate, abrupt, and permanent changes in the level of the outcome variable. However, researchers are confronted with delayed, gradual, and temporary treatment effects; in general, with “response functions” that are markedly different from single-step functions. We here introduce a general framework that allows specifying a test statistic for a randomization test based on predicted response functions that is sensitive to a wide variety of data patterns beyond immediate and sustained changes in level: different latencies (degrees of delay) of effect, abrupt versus gradual effects, and different durations of the effect (permanent or temporary). There may be reasonable expectations regarding the kind of effect (abrupt or gradual), entailing a different focal data feature (e.g., level or slope). However, the exact amount of latency and the exact duration of a temporary effect may not be known a priori, justifying an exploratory approach studying the effect of specifying different latencies or delayed effects and different durations for temporary effects. We provide illustrations of the proposal with real data, and we present a user-friendly freely available web application implementing it.

## Introduction

Single-case experimental designs (SCEDs) are useful for obtaining scientific evidence regarding functional relations and informing professional practice in several fields such as education (Kennedy, 2005), special education (Horner et al., 2005), clinical psychological (Morley, 2018), behavioral sciences (Ledford & Gast, 2018), rehabilitation (Tate & Perdices, 2019), and a variety of other applied settings (Kazdin, 2020, 2021).

The current text focuses on SCED data analysis and, more specifically, on one data analytical option: randomization tests. In the following section, we first review several reasons why the data analysis of SCEDs is not a closed chapter, but a field requiring further research. We also provide a justification for the focus on the paper. Second, we discuss in more detail some features of randomizations tests relevant for the current proposal. Third, we review the prototypical patterns of effect that are to be represented by the effect sizes. Fourth, we refer to possible variations in the operational definitions of: (a) latency in delayed effects; and (b) the abrupt or gradual nature of the effect; and (c) the duration of the effect for temporary effects. Fifth, we present the proposed definition of the test statistic. Finally, we illustrate the use of our proposals in the context of real datasets exhibiting different patterns.

## Rationale

### Lack of consensus regarding data analysis

For analyzing data collected using SCEDs, several quantitative approaches exist and they have already been discussed extensively in the methodological literature (see Busse et al., 2015; Chen et al., 2015; Gage & Lewis, 2013; Manolov & Solanas, 2018; Parker et al., 2011). Similarly, the main advantages and disadvantages of nonquantitative visual approaches have also been presented multiple times (Lane & Gast, 2014; Ledford et al., 2018, 2019; Maggin et al., 2018). However, even in the presence of institutional guidelines regarding quantification of effects, there is no consensus regarding the optimal approach to data analysis. For instance, the What Works Clearinghouse (2022) recommends the design-comparable effect size. The original version of the design-comparable effect size by Hedges et al. (2012, 2013) uses moment estimation and assumes lack of trend, whereas the version suggested as preferential by the What Works Clearinghouse (2022) is the one by Pustejovsky et al. (2014), using restricted maximum likelihood estimation and allowing for trends. The Hedges et al. version has been criticized for disregarding trend, and both version can be criticized of requiring necessarily several participants to be computed, have been explicitly discussed (Kratochwill et al., 2021; Maggin et al., 2022). In the context of other methodological guidelines (e.g., Ganz & Ayres, 2018; Maggin et al., 2014; Tate et al., 2013; Wendt & Miller, 2012) not imposing a single best data analytical option, a common suggestion is to combine visual and statistical analysis and to provide justification for the chosen approach (Fisher et al., 2003; Harrington & Velicer, 2015; Houle, 2009; Kazdin, 2020).

### Limitations of existing approaches

All data analytical techniques have limitations. For instance, nonoverlap indices have a ceiling effect, which entails that they may not distinguish between different magnitudes of intervention effect once the ceiling has been reached (Carter, 2013). Some regression-based approaches also do not discriminate well between different magnitudes of effect (Parker & Brossart, 2003). As another example, the between-case standardized mean difference (Hedges et al., 2012, 2013) and the log-response ratio (Pustejovsky, 2018) assume a lack of trend. The within-case standardized mean difference is affected by potentially irrelevant procedural details of the study’s design (Pustejovsky, 2019), and the interpretation of its values may depend on whether the aim is to increase or reduce the target behavior (Richman et al., 2022). Referring to another data analytical option, multilevel models require a minimum number of participants and measurements to ensure the statistical properties of the inferential information they provide (e.g., Ferron et al., 2009, 2010; Moeyaert et al., 2017). However, with the current text, we do not aim to substitute all other data analytical methods; we are rather aiming to expand the potential of the randomization test approach and introduced next.

### Main features of the randomization test approach

The usefulness of randomization tests in the SCED context was initially suggested by Edgington (1967, 1996). Randomization tests yield *p* values for intervention effects without referring to theoretical sampling distributions. These *p* values allow making a statistical decision regarding the null hypothesis that the intervention is not effective (that is, that the independent and the dependent variables are uncorrelated). Randomization tests have several advantages in the SCED context (Craig & Fisher, 2019; Jacobs, 2019; Kratochwill & Levin, 2010; Onghena, 1992), including the applicability to all kinds of SCEDs^1^, the absence of parametric assumptions^2^, and the flexibility in choosing the test statistic that quantifies the intervention effect. The inference in the context of randomization tests is a tentative causal inference, on the basis of the features of the design and the probability of obtaining a test statistic as extreme as or more extreme than the one actually obtained, in case the null hypothesis of no intervention effect were true. Thus, it is not a population inference, which may not be warranted or of interest when following an idiographic approach (Jacobs, 2019; Onghena et al., 2019).

In terms of their statistical operating characteristics, randomization tests control type I error rates, but the statistical power may not be sufficient for detecting smaller effect sizes (Bouwmeester & Jongerling, 2020; Ferron & Onghena, 1996; Ferron & Sentovich, 2002; Ferron & Ware, 1995; Michiels & Onghena, 2019). It should be noted that the statistical power also depends on the series’ length and the test statistic used (Levin et al., 2021; Michiels et al., 2018), as well as on the randomization scheme (Levin et al., 2018; Manolov, 2019). Another problem with randomization tests is that researchers might be tempted to exclusively focus on *p* values, while the drawbacks and abuses of “*p* value-driven” statistical analysis have been extensively documented and debated (Wasserstein & Lazar, 2016). However, the *p* values (generated by a randomization test or otherwise) should never be interpreted in isolation, but rather be considered as additional information to descriptive statistical measures, an assessment of the effect magnitude, a graphical display of the interrupted time series, and a judgement about the clinical significance or the practical relevance of the obtained effects (or the absence of these effects). Furthermore, in a more comprehensive “randomization test approach” equal attention should be paid to the definition, calculation, and interpretation of the test statistic that is used to quantify the intervention effect (Heyvaert & Onghena, 2014a), to the importance of randomization for excluding confounding factors and for deriving a valid probabilistic statement (Edgington, 1996), and to the possibility of test inversion for deriving randomization-based confidence intervals (Michiels et al., 2017).

In terms of the conditions for application, the valid use of randomization tests depends on two complementary requirements. One requirement is to actually use randomization in the design, which is a desirable methodological feature (Kratochwill & Levin, 2010; Tate et al., 2013). Moreover, as stated by Levin et al. (2018), the presence of randomization in the design is necessary for the exchangeability assumption that each value in the randomization distribution is equally likely to have occurred in the absence of an intervention effect given that in the absence of randomization, this assumption may not be tenable if there is trend and/or autocorrelation in the data. Another requirement is to perform the data divisions (for obtaining the reference distribution) in a way consistent with the random assignment procedure (Edgington, 1980a, 1980b). With the term “data division”, we refer to the specific way in which the series of measurements, whose order is maintained fixed, as actually obtained, is divided into different conditions (e.g., baseline and treatment). For instance, in the context of a multiple-baseline design, it is possible to decide at random for each tier^3^ when the intervention begins, out of a set of possible values, and also to decide at random for which tier the intervention is introduced first (see the Koehler–Levin procedure; Koehler & Levin, 1998; Levin et al., 2018). In such a case, the data divisions^4^ to be performed once the data are gathered should mirror this randomization scheme and not, for instance, be performed as if there were only a random order of tiers and a fixed intervention start point for each tier (i.e., Wampold–Worsham procedure; Levin et al., 2018; Wampold & Worsham, 1986).

One noteworthy feature of randomization tests is that the effect size measure (to which a *p* value is later associated) is to be chosen prior to gathering the data according to the type of intervention effect expected (Ferron & Ware, 1995; Heyvaert & Onghena, 2014b). Although choosing (and reporting publicly) the data analytical approach before data collection has recently been emphasized in the context of SCEDs (Johnson & Cook, 2019; Manolov et al., 2022; Porcino et al., 2020), this emphasis is characteristic in relation to randomization tests for SCEDs (Edgington, 1975, 1980b). This historical emphasis in the context of randomization tests has to be considered in light of the traditional use of response-guided experimentation and visual analysis (as reviewed Lane & Gast, 2014; Ledford et al., 2019; Maggin et al., 2018), which entails repeatedly assessing multiple data features such as level, trend, immediacy, and overlap (highlighted by Parker et al., 2006, as a strength of visual analysis) in the course of data collection, regardless of any initial expectations. With randomization tests it is possible to accommodate several data patterns (e.g., change in trend, change in variability; Levin et al., 2021), whereas the other data analytical methods are mainly applicable to immediate effects (change in level, as in the BC-SMD, or change in slope as in the multilevel models). In the context of this flexibility and versatility, we here propose a general framework for defining the test statistic for different possible data patterns.

## A unified framework: Response functions

### Test statistics used in the SCED literature

Randomization tests can be devised for any test statistic that is chosen prior to looking at the data; the test statistic should represent the expected experimental effect as closely as possible to have a sensitive test (Ferron & Ware, 1995; Heyvaert & Onghena, 2014b). Several possible test statistics have been studied for specific expected effects, including quantifications of immediate effects (Michiels & Onghena, 2019) and delayed effects (Levin et al., 2017). In terms of the focal data feature and the test statistic, level differences (e.g., using means) have been most frequently discussed and/or investigated (e.g., Edgington, 1975; Ferron & Ware, 1995; Onghena, 1992), which is consistent with level being commonly the focus of the data analysis in the SCED context (Tanious & Onghena, 2021). A mean difference would be a sensible choice when stable baseline data are expected and the effect is expected to be abrupt and sustained.

However, all visually analyzed data features (Kratochwill et al., 2013) have been discussed and/or tested as test statistics. Specifically, it is possible to quantify (as a test statistic) a change in slope and a change in variability (Levin et al., 2021), nonoverlap (Heyvaert & Onghena, 2014a), and consistency (Tanious et al., 2019). A change in slope could be the choice of a test statistic when a progressive linear effect is expected, whereas a nonoverlap index in case the measurement of the target variable is expressed in ordinal terms and an interval or ratio scale cannot be assumed.

The flexibility in choosing a quantification of the magnitude of effect corresponds to the fact that the randomization test can include any complex statistic (Edgington, 1980b). For instance, the main quantification could stem from a statistical model such as multilevel models, with the randomization test being used for obtaining the *p* values (Michiels et al., 2020). Finally, different test statistics are possible according to the kind of SCED. For instance, a quantification of the distance between data paths has been suggested as a test statistic in alternating treatment designs (Manolov, 2019), and a quantification of the distance between the predefined criterion level and the actual measurements has been suggested as a test statistic in a changing criterion designs (Onghena et al., 2019).

### What the test statistic should represent: Prototypical patterns of intervention effects

There are several possible kinds of treatment effect. In terms of the time dimension, the effect can be immediate or delayed (Riley-Tillman et al., 2020; Tate & Perdices, 2019); temporary or permanent (Houle, 2009). In terms of the way in which the final behavioral level is reached, the effect can be abrupt or gradual (Levin et al., 2021; Swan & Pustejovsky, 2018). Moreover, it is possible to combine these different patterns of effect, which would lead to the following prototypes:

1. An immediate and abrupt change that is permanent. This could be understood as the simplest kind of effect, representing also what is quantified in the between-case standardized mean difference (or design-comparable effect size) as described in Hedges et al. (2012, 2013) and Shadish et al. (2014). The aim of presenting a more general framework is to offer analytical options beyond this data pattern.
2. An immediate and abrupt change that is temporary
3. An immediate and gradual change that is permanent
4. An immediate and gradual change that is temporary
5. A delayed and abrupt change that is permanent
6. A delayed and abrupt change that is temporary
7. A delayed and gradual change that is permanent
8. A delayed and gradual change that is temporary

**Fig. 1:**
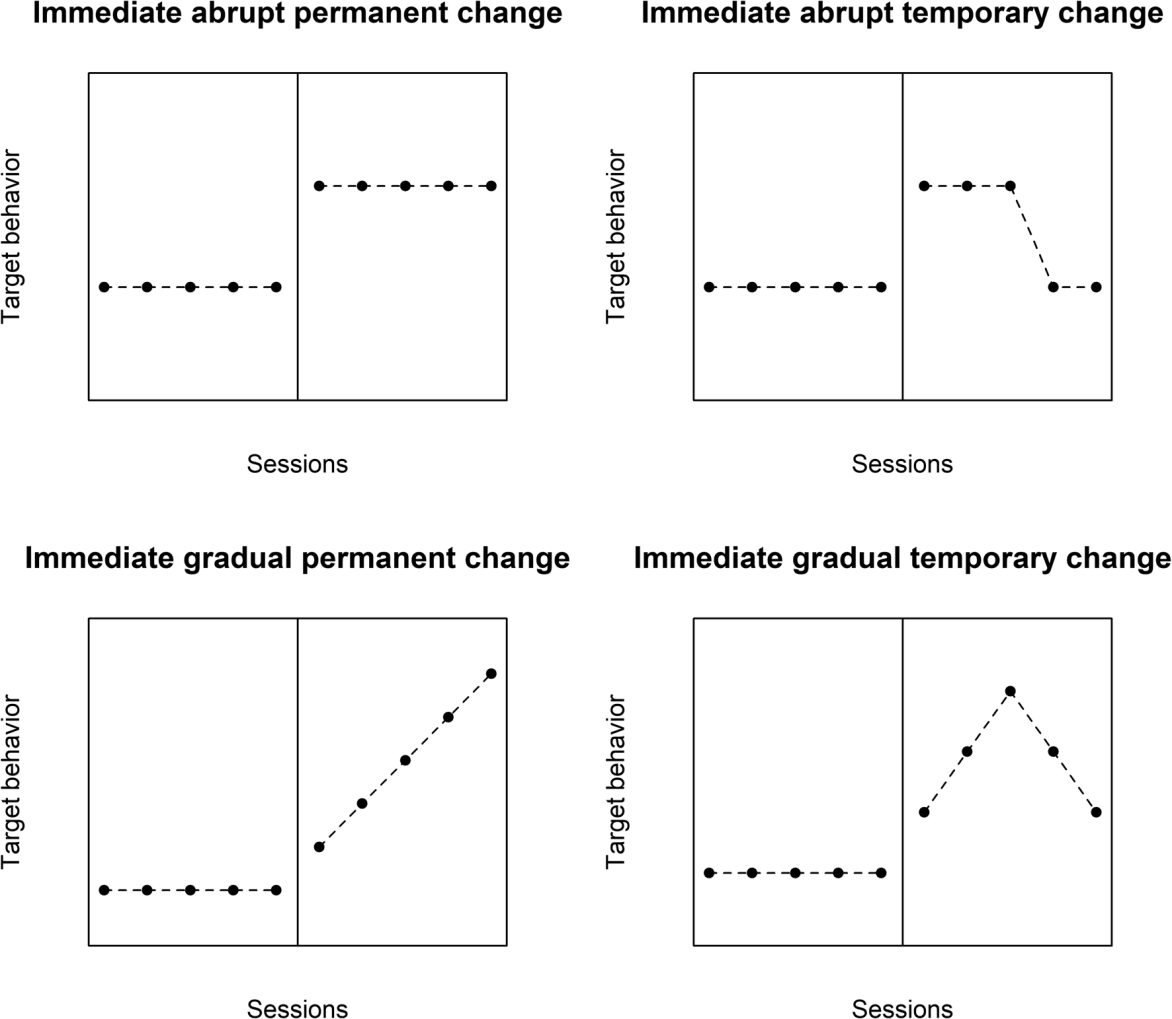
Prototypical immediate effects

**Fig. 2:**
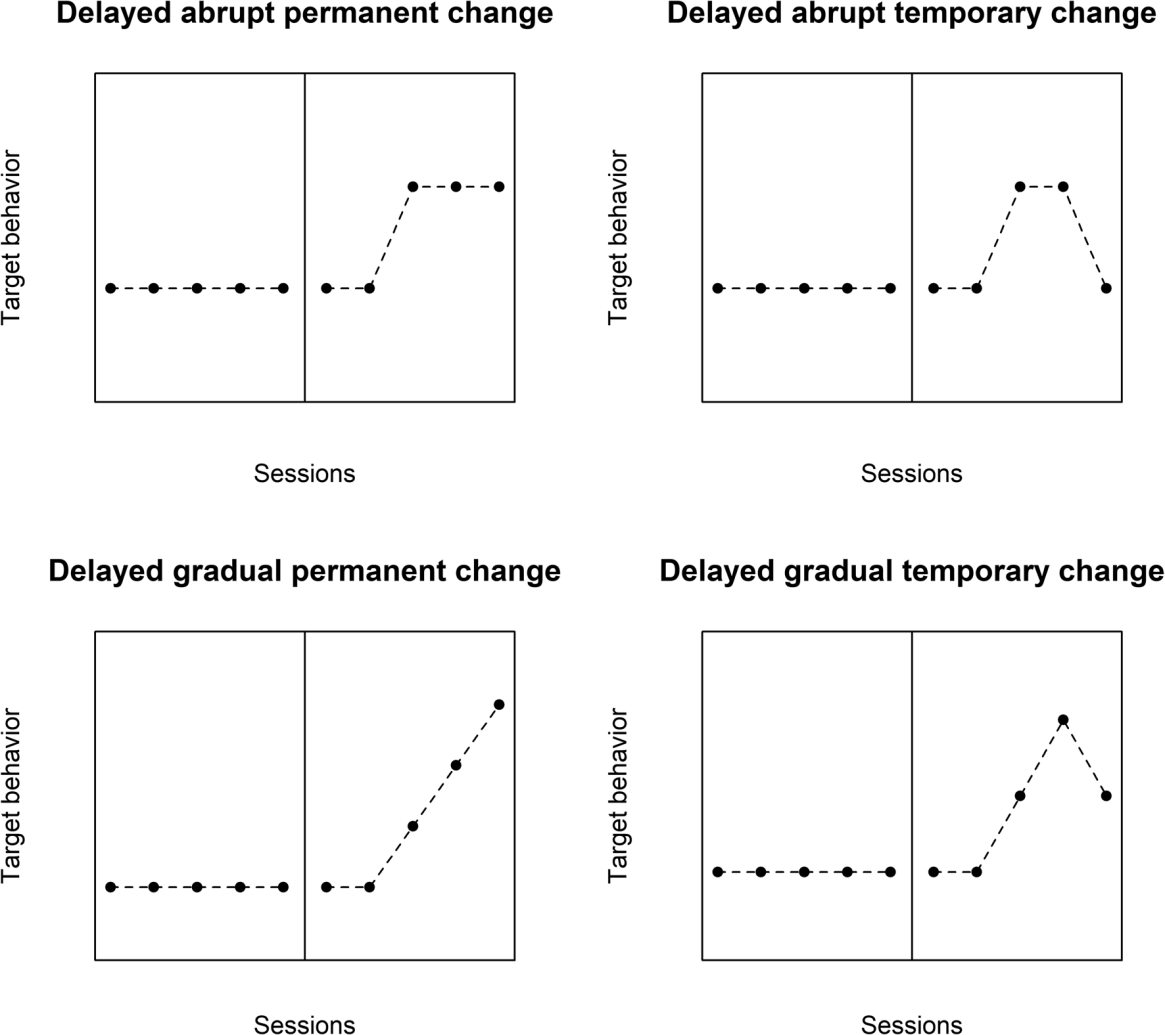
Prototypical delayed effects

The immediate effects are depicted on Fig. 1, whereas the delayed effects are depicted in Fig. 2. These figures are simplifications, representing only one possible duration of the temporary effects and one possible latency. On the one hand, the duration of the delay or latency (i.e., after how many intervention phase measurement occasions does the effect begin) and the duration of the temporary effect (i.e., after how many intervention phase measurement occasions does the effect begin to disappear or wear off) can be specified. On the other hand, these two aspects can be incorporated in an exploratory approach (i.e., checking the consequences of varying the latency and varying the duration of the effect). Similarly, note that, just like there are different ways in which the intervention effect can appear, there are also different ways in which it can disappear: in an abrupt or gradual way. Finally, these patterns represent situations in which the aim is to increase the target behavior, but the current text (and the later proposal of “response functions”) is applicable to the desired change being either an increment or a reduction.

An additional comment is warranted on the variety of data patterns depicted in Figs. 1 and 2. In some situations, effects are desired to be permanent, in the sense that the gains are not lost with time. This agrees with the assessment of maintenance in the context of social validation (Snodgrass et al., 2018). Nevertheless, a progressive effect (e.g., an improving trend during the intervention phase) could be expected to reach an asymptote, as no further improvement is possible, and such a data pattern would not suggest that the effect has been lost with time. In that sense, it is relevant whether a temporary effect is conceptualized as “disappearing” or as “not showing further improvement”. Moreover, in some situations (e.g., when the target behavior is an emotion or a secondary effect of a medicine), a temporary effect as depicted in the upper right panel of Fig. 1 may be expected or even desired. Additionally, delayed effects may still be tolerated when there is an expectation (and previous evidence) of a transition state or an extinction burst (Brogan et al., 2019; Katz & Lattal, 2021). Finally, a delayed and abrupt change that is temporary (upper right panel of Fig. 2) may not be considered convincing, unless there is a clear expectation on the basis of theory or previous research about such a data pattern.

### Exploratory approach in the context of the prototypical effects

In the context of several possible prototypical effects, we are not suggesting a completely ad hoc exploration of all possibilities. Instead, we consider that existing theoretical knowledge and empirical evidence regarding the research domain (including type of intervention and features of the target behavior) should be used when making explicit the expectations regarding the potential delay, progression, and duration of the effect. Specifically, when referring to delayed effects, in order to reduce the probability of confirmation bias, the amount of latency has to be defined beforehand, instead of being the result of the visual inspection of the graphed data. Similarly, for temporary effect, the duration of the effect (expressed as the number of measurement occasions) has to be defined before gathering and analyzing visually the data. In case there is no sound reason^5^ why a given number of values should be considered as the obvious choice, an option is to try out different latencies and different durations and check how the results differ under these different operational definitions of delayed and temporary effects. Such an exploratory approach is reasonable in case there is no specific delay or duration that has already been reported in the literature and is intended to be confirmed. Finally, the *p* values should be corrected for multiple testing if they are used as probabilistic statements. In contrast, in a pure exploratory approach, the *p* values can also be used as mere descriptive measures.

### Defining the response function and incorporating the exploratory approach

We refer to the general framework for representing different kinds of effects as using “response functions”. A response function is a vector of constants describing the predicted response. Thus, the specific choice of a response function would depend on the kind of effect expected.

We present the idea in the context of a simple example of a hypothetical design with five baseline measurements and five intervention phase measurements, representing the number of aggressive behaviors performed by a child on the playground. We first illustrate the use of a response function for the eight prototypes.

1. An immediate and abrupt change that is permanent: the response function would be {0, 0, 0, 0, 0, 1, 1, 1, 1, 1} (see upper left panel of Fig. 1). Such a response function would include all baseline data and all intervention phase data, as the expectation is for the effect to continue until the end of the phase.
2. An immediate and abrupt change that is temporary: the response function could be {0, 0, 0, 0, 0, 1, 1, 1, 0, 0} (see upper left right of Fig. 1). In this case, given that the expectation is for the effect to be only temporary, the last intervention phase measurements are coded differently. Specifically, “temporary” is here arbitrarily defined as referring to the first three intervention phase data points and it disappears in an abrupt way. Here we can explore a different number of measurement occasions during which the immediate effect lasts (e.g., 1–5).
3. An immediate and gradual change that is permanent: the response function could be {0, 0, 0, 0, 0, 1, 2, 3, 4, 5}, representing an immediate change in slope (Levin et al., 2021; Wampold & Furlong, 1981). For a graphical representation, see the lower left panel of Fig. 1. The idea is that the specific values of the response function for a gradual change depend on the length of the intervention phase (i.e., when there are *n*_*A*_ baseline phase measurements and *n*_*B*_ intervention phase measurements, the value 0 is repeated *n*_*A*_ times, followed by integers from 1 to *n*_*B*_). As explained below, nonlinear gradual effects can also be incorporated in the response function.
4. An immediate and gradual change that is temporary: the response function could be {0, 0, 0, 0, 0, 1, 2, 3, 2, 1} (see lower right panel of Fig. 1). Just like before, “temporary” is here arbitrarily defined as referring to the first three intervention phase data points and it disappears in the same gradual way in which it appeared. Once again, an exploratory approach can be used here in defining the duration of the effect, as expressed in the number of sessions.
5. A delayed and abrupt change that is permanent: it is possible to represent a delayed change in level by omitting some of the initial intervention phase values (Levin et al., 2017; see also Brogan et al., 2019, for a similar suggestion when discussing transition states). Another option is to use a quantification such as the “mean baseline reduction” that focuses only on the last three measurements of each phase (Olive & Smith, 2005). In order not to discard any baseline data, the response function for a delayed effect could be {0, 0, 0, 0, 0, 0, 0, 1, 1, 1} (see upper left panel of Fig. 2). This specific coding reflects an expectation that the onset of the effect will be after two intervention phase measurement occasions. Another option is to explore several different possible delays or latencies.
6. A delayed and abrupt change that is temporary: the response function could be {0, 0, 0, 0, 0, 0, 0, 1, 1, 0} (see upper right panel of Fig. 2). In this specific example, there is again a delay of two measurement occasions, and after two more, the effect of the intervention disappears. The exploratory approach can be applied both to the latency and to the duration of the effect.
7. A delayed and gradual change that is permanent: the response function could be {0, 0, 0, 0, 0, 0, 0, 1, 2, 3} (see lower left panel of Fig. 2). The exploratory approach can again be applied to the latency of the effect.
8. A delayed and gradual change that is temporary: the response function could be {0, 0, 0, 0, 0, 0, 0, 1, 2, 1} (see upper left panel of Fig. 2). In this specific example, there is again a delay of two measurement occasions, and after two more, the effect of the intervention disappears. The exploratory approach can be applied both to the latency and to the duration of the effect.

It should be noted that there are several options for defining a response function to represent each of the effects, whose ideal versions are shown in Figs. 1 and 2. For instance, it is possible to represent an intervention effect that reaches an upper asymptote (e.g., using the response function {0, 0, 0, 0, 0, 1, 2, 3, 3, 3}). Additionally, the response functions can be adapted to accommodate for the expectation of a general linear trend. This can be achieved by adding 1, 2, …, *n* (where *n* is the number of measurements in the baseline and intervention phases being compared), to the previously presented response functions. Both of these options have been incorporated in the software developed (described later). Moreover, the default options of the software can be overruled by the user, by means of specifying user-defined functions.

From these eight examples, it is easy to infer how the constants have to be modified to model other experimental effects, including nonlinearity and gradual decay. For instance, a response function such as {0, 0, 0, 0, 0, 2, 4, 8, 16, 32} can be used for representing a kind of nonlinear trend.

### Pearson’s correlation coefficient as a quantification of effect

The null hypothesis of the randomization test (i.e., no effect of the intervention) can be understood as the independent and the dependent variable being uncorrelated. In that sense, a general test statistic should be sensitive to deviations from this null model. Accordingly, if we want to quantify the linear association between the observed data and the predicted response function, then Pearson’s product-moment correlation coefficient is a common choice (see e.g., Chapters 8 and 9 on Correlation and Trend Tests in Edgington & Onghena, 2007). Note that it is not a problem if the response function only consists of 0s and 1s. If Pearson’s correlation coefficient is used for quantifying the relation between a quantitative variable (e.g., the measurements of the target behavior) and a binary variable (e.g., the response function for an immediate and abrupt effect that is permanent), it is called the point-biserial correlation coefficient. Using this coefficient is equivalent to computing a mean difference in terms of the *p* value that would be obtained from the corresponding statistical test (Edgington & Onghena, 2007; Ruscio, 2008). In other words, the correlation coefficient can be used in the general case, whereas the difference between means only in the special case (if one of the variables is binary). Note that the fact that Pearson’s correlation is a quantification of a linear relation; it does not mean that the data pattern should be linear because the data pattern is represented by the response function. For instance, in case the measurements were {3, 3, 3, 3, 3, 7, 13, 25, 49, 97}, and the response function was defined as {0, 0, 0, 0, 0, 2, 4, 8, 16, 32}, the value of Pearson’s correlation coefficient would be 1.

Even though we here propose using the correlation coefficient as a quantification of the magnitude of effect and also as a test statistic, researchers are free to compute an additional quantification of effect, according to the data pattern expected. Examples of such quantifications include: (a) for the data pattern on the top left panel of Fig. 1, a mean difference; (b) for the data pattern on the bottom left panel of Fig. 1, a slope difference; (c) for the data pattern on the top left panel of Fig. 2, a mean difference, excluding the initial measurements for the intervention phase (as suggested in Levin et al., 2017); and (d) for the data pattern on the bottom left panel of Fig. 2, a mean difference, excluding the initial measurements for the intervention phase.

### Pearson’s correlation coefficient as a test statistic in a randomization test

Once the observed value of Pearson’s correlation coefficient is computed, it can be located in the randomization distribution, constructed according to the randomization scheme for selecting the point of change in phase. For instance, if there are 15 measurement occasions and a minimum of three measurement occasions per phase, the intervention can be chosen (at random) to start anywhere between measurement occasions 4 and 13, both inclusive. This would lead to ten possible data divisions. Once the random selection of the intervention start point is performed, the data are gathered. Afterwards, the test statistic is computed for all (for instance, ten) admissible data divisions. The randomization distribution would contain ten values of the test statistic: the value for the actual data division (called “the observed value of the test statistic”) and the values for the remaining possible data divisions (called “pseudovalues” or “potential values”). These ten values are ordered. Afterwards, a rank or a *p* value can be assigned to the statistic.

### Exceptional results

Carrying out a randomization test entails a quantification of the degree to which the value of the test statistic chosen is exceptional if the null hypothesis of no intervention effect is true. On the basis of the randomization in the design, all possible divisions of the data (i.e., moments of phase change) are equally likely under the null hypothesis. Thus, the actually obtained value for Pearson’s correlation coefficient is compared to the correlation coefficient values that would have been obtained for all the admissible points of change in phase. The aim is to quantify the degree to which the former is expected to happen in absence of an effect. If the probability is small (e.g., equal to or smaller than a predefined value such as .05), then the results are considered “statistically significant”. That is, such a large value of *r* would be unexpected if there was no treatment effect.

## Illustrations of the use of response functions

In the current section, we will illustrate the use of response functions and Pearson’s correlation coefficient as a test statistic, along with the possibility of applying an exploratory approach. The data for the illustration were gathered by te Brake et al. (2023), studying the effect of beat frequency to adjust running cadence (steps per minute) in recreational runners, with the aim of increasing this target behavior. A multiple-baseline design across participants was used and the seven participants were randomly assigned, following the Wampold–Worsham procedure (Levin et al., 2018; Wampold & Worsham, 1986) to one of the seven possible baseline lengths. Specifically, the baselines could last from four to ten measurement occasions, whereas the measurement occasions for the intervention phase were fixed to eight for all participants. According to this randomization procedure, there are 7 !  = 5040 possible randomizations (or orders of the seven participants). For the current illustration, we will focus on three of the seven participants separately (i.e., studying each A-B comparison individually), considering that their baseline had a minimum length of four and a maximum of ten. We will use different response functions to show how different data patterns (potentially present in the data for the different participants) can be accommodated. Nevertheless, in an actual multiple-baseline study, in case the participants have similar features, and their target behaviors and interventions are identical, it would be logical to use the same response function for all participants, assuming that the expectation about the data pattern is the same for all of them.

### Immediate permanent or temporary change in level (abrupt effect)

**Fig. 3:**
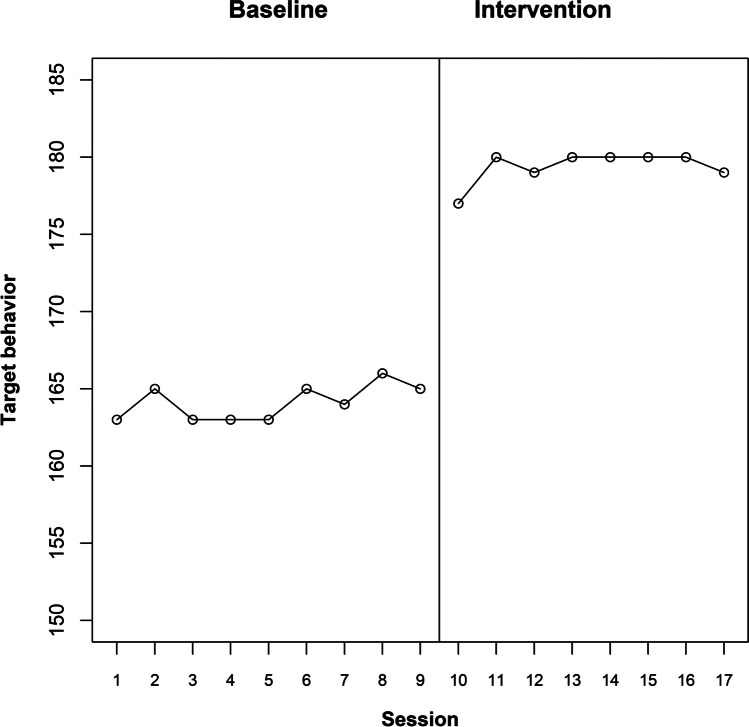
te Brake et al. (2023) data for participant 4

Figure 3 represents the raw data for participant 4, suggesting that there is a clear immediate change in level, which lasts until the end of the intervention phase. As an initial illustration, we will suppose that the expected effect is an immediate, abrupt, and permanent change in level. In this case, the response function is a binary variable containing as many 0s as baseline measurement occasions and as many 1s as intervention phase measurement occasions. Beyond the specific features of the data depicted in Fig. 3, we could also check whether the effect could be temporary, exploring immediate abrupt effects that last between one and five sessions. For representing these temporary effects via response functions, the number of 1s is going to be one, two, three, four, or five, according to the specified duration of the effect. These 1s are preceded by as many 0s as baseline measurement occasions and they are also followed by 0s, until the end of the intervention phase. The response functions for the actual point of change in phase for participant 4 are included in Table 1. Note that for each admissible intervention start point, the number of baseline 0s and the number of intervention phase 1s and 0s varies according to the length of the phases.

**Table 1:** Response functions for the te Brake et al. (2023) data for participant 4, representing immediate abrupt effects: permanent and temporary

Session	Phase	Immediate abrupt permanent	Immediate abrupt temporary 1	Immediate abrupt temporary 2	Immediate abrupt temporary 3	Immediate abrupt temporary 4	Immediate abrupt temporary 5
1	Baseline	0	0	0	0	0	0
2	Baseline	0	0	0	0	0	0
3	Baseline	0	0	0	0	0	0
4	Baseline	0	0	0	0	0	0
5	Baseline	0	0	0	0	0	0
6	Baseline	0	0	0	0	0	0
7	Baseline	0	0	0	0	0	0
8	Baseline	0	0	0	0	0	0
9	Baseline	0	0	0	0	0	0
10	Intervention	1	1	1	1	1	1
11	Intervention	1	0	1	1	1	1
12	Intervention	1	0	0	1	1	1
13	Intervention	1	0	0	0	1	1
14	Intervention	1	0	0	0	0	1
15	Intervention	1	0	0	0	0	0
16	Intervention	1	0	0	0	0	0
17	Intervention	1	0	0	0	0	0

**Fig. 4:**
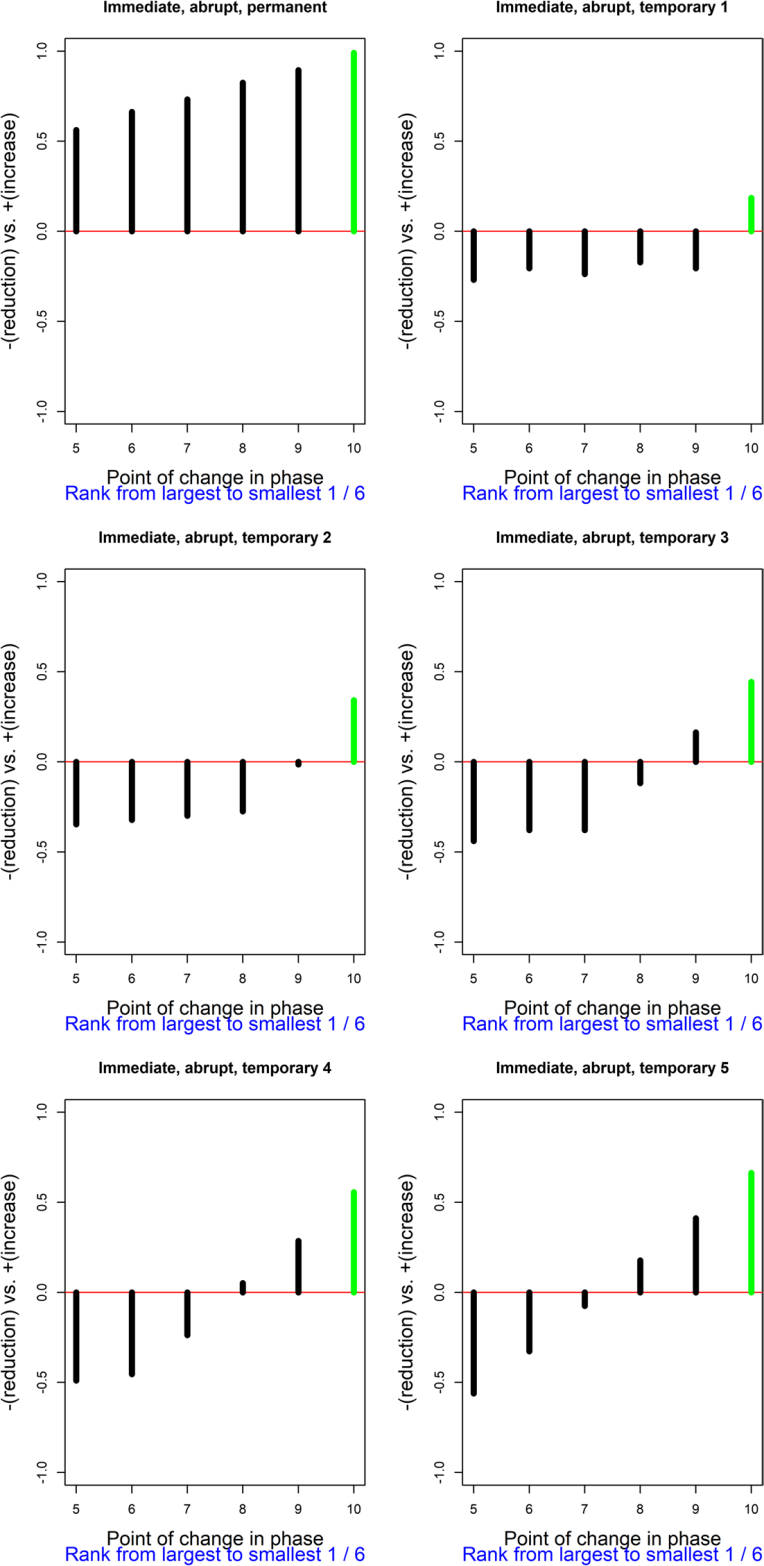
Results of the exploratory randomization approach, using Pearson’s correlation as test statistic, for the te Brake et al. (2023) data for participant 4. Exploring an immediate and abrupt effect

The result of using Pearson’s correlation coefficient as a test statistic, quantifying the relation between the response function and the measurements of the target behavior, is depicted in Fig. 4. In this plot, each admissible point for change in phase is represented on the abscissa (*X*-axis). On the ordinate (*Y*-axis), the value of Pearson’s correlation for the actual point of change in phase and for all admissible points of change in phase are represented. The *Y*-axis ranges theoretically between − 1 and + 1. A horizontal line is included for 0. Positive values (above the horizontal line) are “favorable” results; that is, results in line with the expected effect or the alternative hypothesis (here, a reduction of the target behavior). In general, regardless of whether the desired result is an increase or a decrease of the target behavior, if the actual result agrees with the desired one, it would be represented above the horizontal line. Furthermore, the value of the actual test statistic is depicted in green, given that a favorable result is obtained. (In case an unfavorable result were obtained, it would have been depicted in red.) For the results represented in Fig. 4, the most salient aspect is that the current data division is associated with the largest value of the test statistic, regardless of whether the immediate abrupt effect is considered as permanent or temporary. Considering that the value of Pearson’s correlation coefficient between the response function and the measurements is largest for the actual data division, out of six possibilities, the *p* value would be 1/6 ≈ 0.167, the smallest possible for this A-B comparison. Therefore, the result is consistent with the expectation of an immediate and abrupt effect (regardless of whether it is sustained or temporary). The result is not statistically significant if we compare the *p* value to the commonly used nominal alpha of .05, but such a level of significance cannot be achieved only from a single A-B comparison with few possible intervention start points. In contrast, statistical significance can be achieved when considering all A-B comparisons (i.e., all participants) in the multiple-baseline design. For readers interested in the application of randomization tests to multiple baseline design, we recommend the following articles by Levin and colleagues (Levin et al., 2017, 2018; Levin & Gafurov, 2019). The logic of the randomization to be performed prior to gathering the data and the (same) randomization to be carried out for obtaining the reference distribution would be the same as described in these articles, with the main difference being the test statistic used (which in the current proposal is Pearson’s correlation coefficient, computed using the measurements and the response function as the two variables).

### Immediate gradual effect

**Fig. 5:**
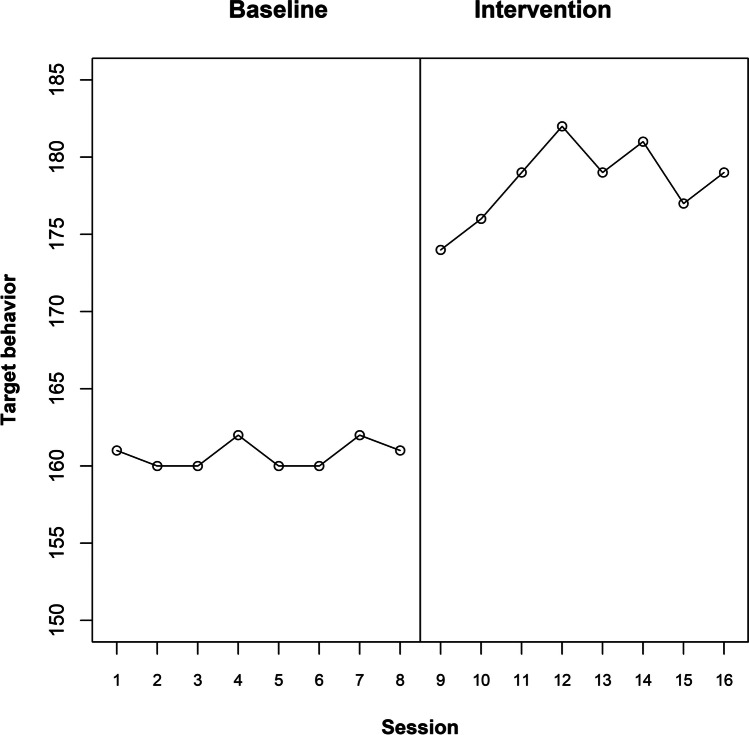
te Brake et al. (2023) data for participant 3

The raw data for participant 3 are depicted in Fig. 5, suggesting an immediate effect, which is gradually increasing. For illustrative purposes, we will suppose that an immediate gradual effect was expected, expressed as a change from a stable baseline to a nonzero slope in the intervention phase.

**Fig. 6:**
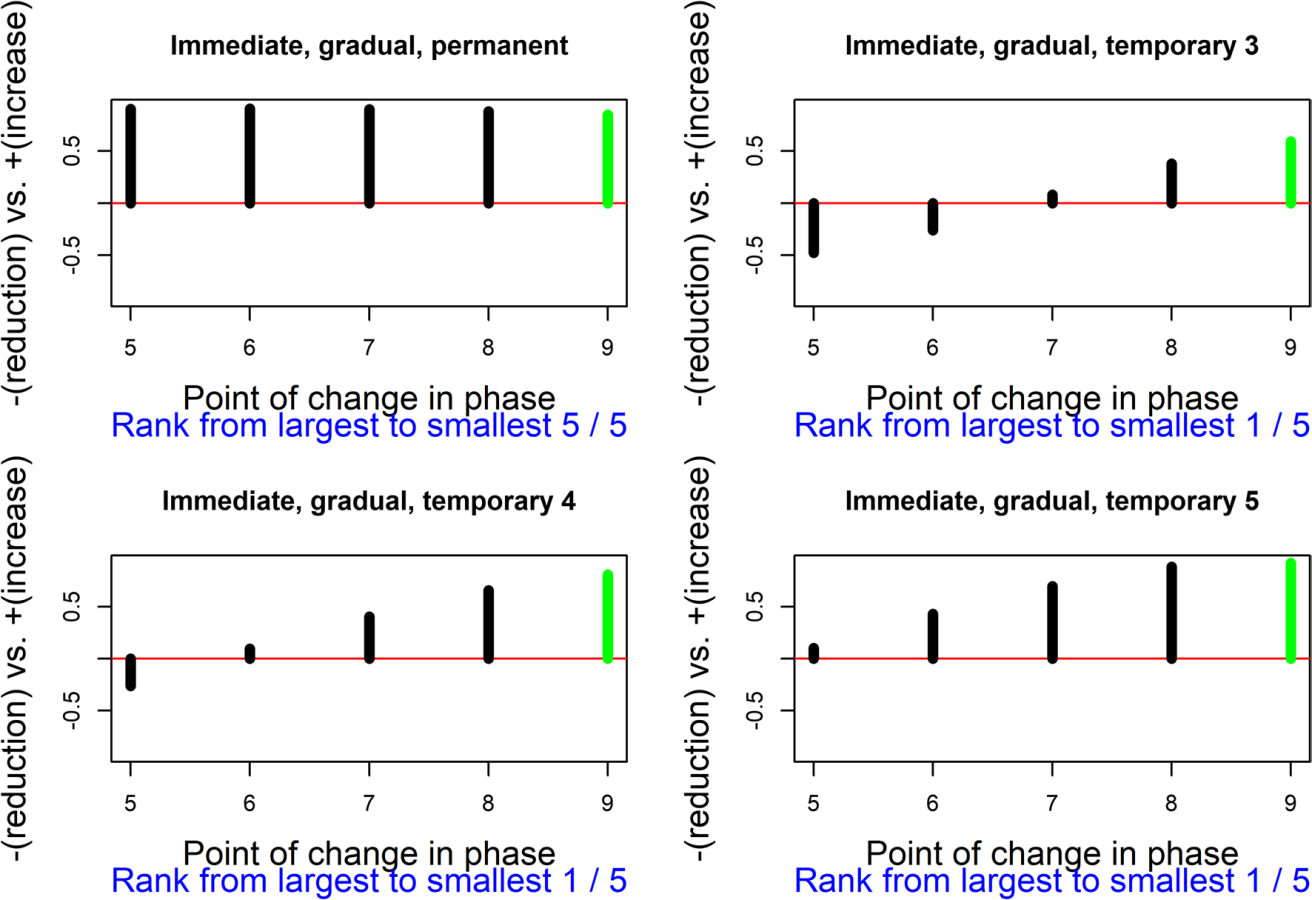
Results of the exploratory randomization approach, using Pearson’s correlation as test statistic, for the te Brake et al. (2023) data for participant 3. Exploring an immediate gradual effect

Via a randomization test, it is possible to quantify the degree to which the observed linear increase of the target behavior in the intervention phase is likely to be obtained in absence of an intervention effect. The response functions for the actual point of change in phase for participant 3 are included in Table 2. Figure 6 represents the results for a permanent and a temporary effect (e.g., a positive slope that disappears gradually). Looking at the practically identical values of Parson’s correlation coefficient, the permanent effect is similarly large for all admissible intervention start points. In consequence, the *p* value is 1, and it suggests that there is no evidence for a gradual effect. This is because once the eight actual intervention phase measurements are included, their higher values (compared to the initial baseline measurements) lead to large differences also for previous potential intervention start points. Actually, if we were looking for an immediate abrupt (rather than gradual effect), the *p* value would have been the smallest possible, 1/5 = 0.20. In contrast to the lack of evidence of an immediate gradual effect sustained in time, the actual intervention start point is associated with the largest gradual increase if only the initial three-to-five intervention phase measurements are considered. This is because the initial intervention phase measurement occasions suggest a progressive effect, whereas in the latter ones apparently an upper asymptote is reached.

**Table 2:** Response functions for the te Brake et al. (2023) data for participant 3, representing immediate gradual effects: permanent and temporary (with a gradual offset)

Session	Phase	Immediate gradual permanent	Immediate gradual temporary 3	Immediate gradual temporary 4	Immediate gradual temporary 5
1	Baseline	0	0	0	0
2	Baseline	0	0	0	0
3	Baseline	0	0	0	0
4	Baseline	0	0	0	0
5	Baseline	0	0	0	0
6	Baseline	0	0	0	0
7	Baseline	0	0	0	0
8	Baseline	0	0	0	0
9	Intervention	1	1	1	1
10	Intervention	2	2	2	2
11	Intervention	3	3	3	3
12	Intervention	4	2	4	4
13	Intervention	5	1	3	5
14	Intervention	6	0	2	4
15	Intervention	7	0	1	3
16	Intervention	8	0	0	2

### Delayed abrupt effect

**Fig. 7:**
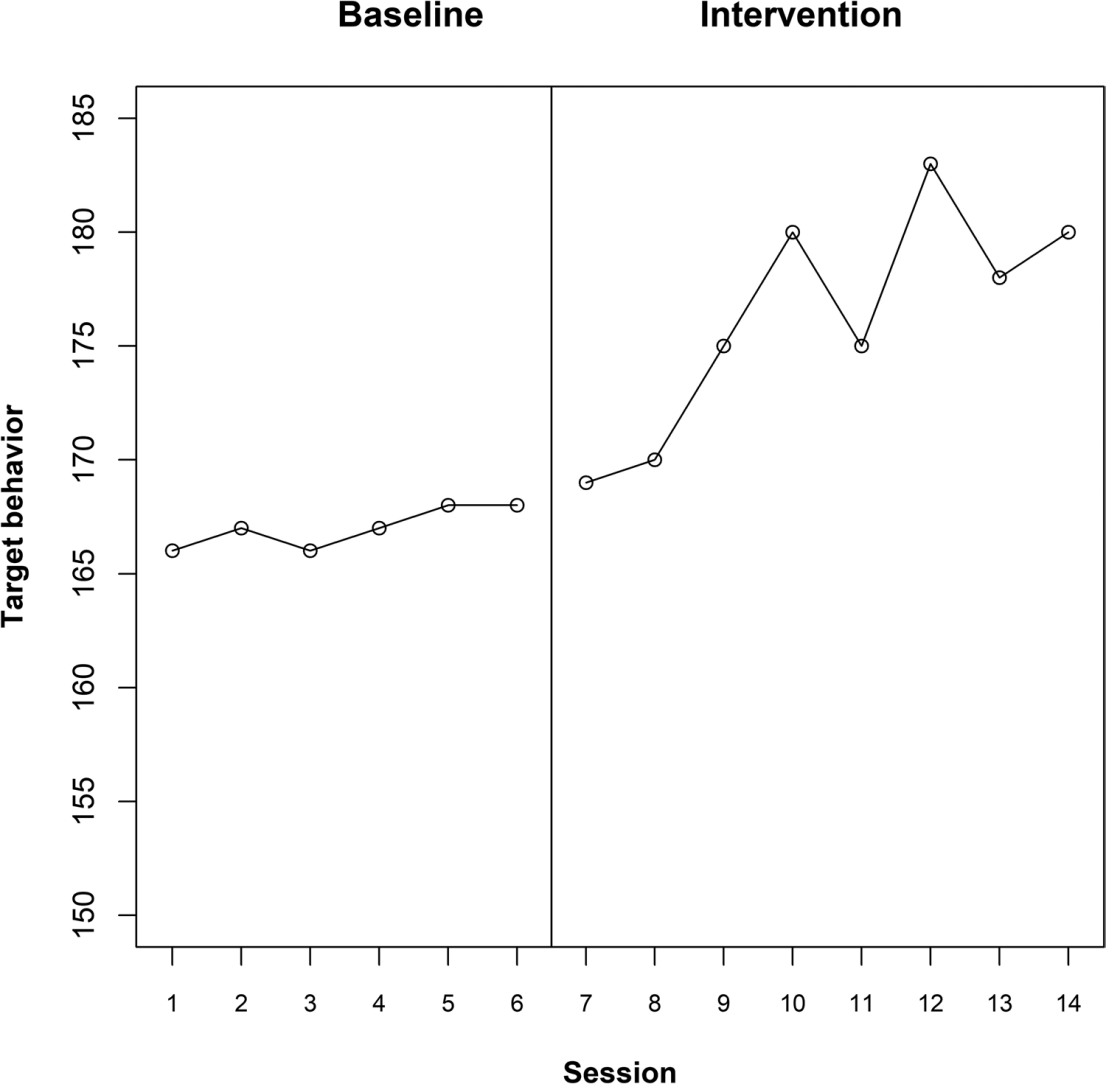
te Brake et al. (2023) data for participant 5

The raw data for participant 5 are depicted in Fig. 7, suggesting that there is either a gradual or a delayed increase of the target behavior in the intervention phase. For illustrative purposes, we will suppose that delayed abrupt change in level was expected.

**Fig. 8:**
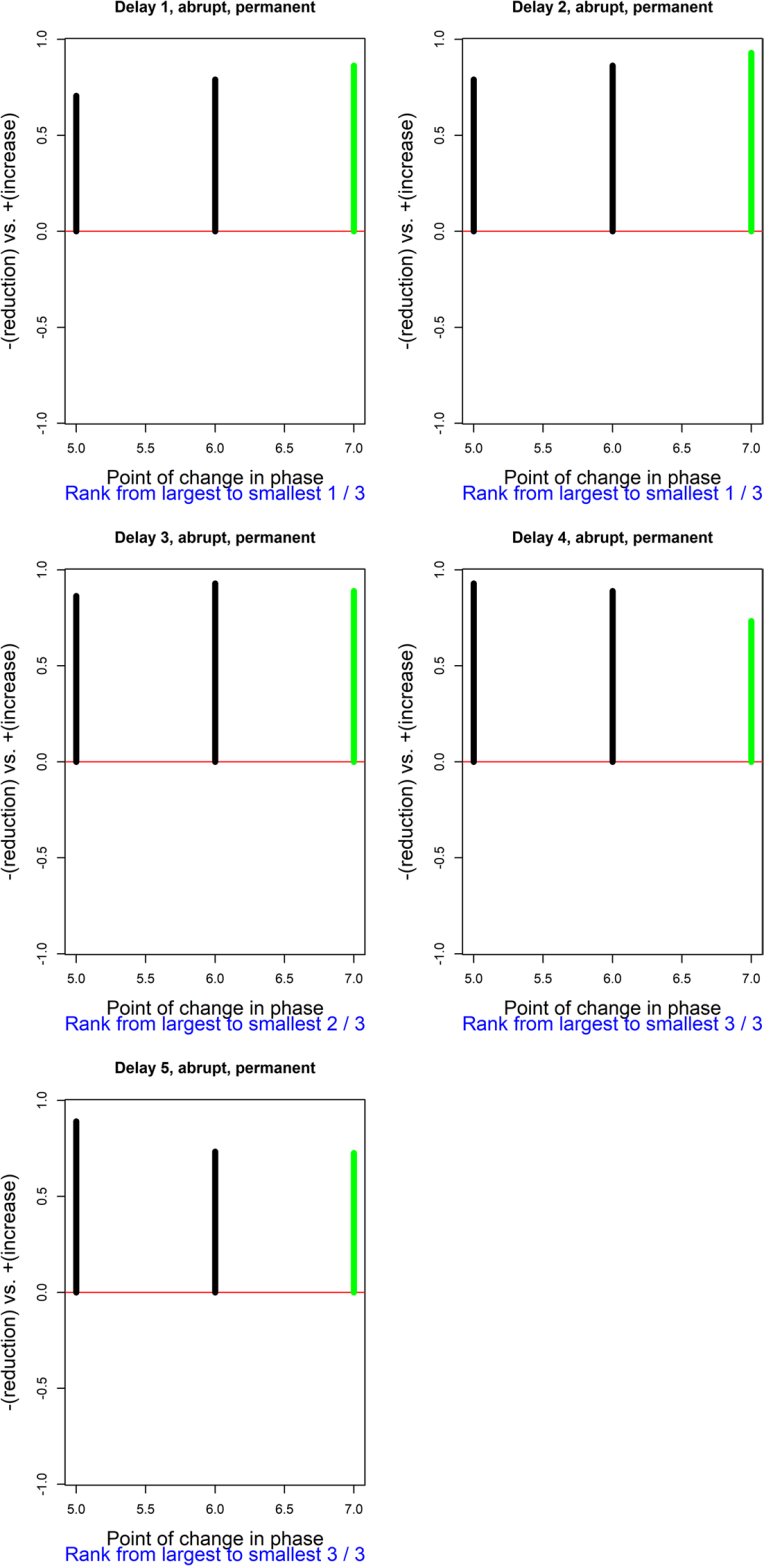
Results of the exploratory randomization approach, using Pearson’s correlation as test statistic, for the te Brake et al. (2023) data for participant 5. Exploring a delayed effect

The response functions for the actual point of change for participant 5 are included in Table 3. Figure 8 represents the results for a delayed effect. The strongest evidence is for a delay of the intervention effect of one or two measurement occasions, which is when the value of Pearson’s correlation coefficient is the largest (i.e., the *p* value is the smallest possible, 1/3 ≈ 0.33). Longer delays are not supported by the evidence. This aligns well with the visual inspection of the data, suggesting that the change in level occurs (or stars being clearer) at the third intervention phase measurement occasion.

**Table 3:** Response functions for the te Brake et al. (2023) data for participant 3, representing a delayed abrupt permanent effect, with a different amount of the delay

Session	Phase	Delayed 1	Delayed 2	Delayed 3	Delayed 4	Delayed 5
1	Baseline	0	0	0	0	0
2	Baseline	0	0	0	0	0
3	Baseline	0	0	0	0	0
4	Baseline	0	0	0	0	0
5	Baseline	0	0	0	0	0
6	Baseline	0	0	0	0	0
7	Intervention	0	0	0	0	0
8	Intervention	1	0	0	0	0
9	Intervention	1	1	0	0	0
10	Intervention	1	1	1	0	0
11	Intervention	1	1	1	1	0
12	Intervention	1	1	1	1	1
13	Intervention	1	1	1	1	1
14	Intervention	1	1	1	1	1

## Software use

The freely available Shiny app (https://manolov.shinyapps.io/ResponseFunction) was created, implementing the proposal for the exploratory randomization approach. The input data file is a simple text file with two columns. One column is called “phase” and it contains the letters A and B, denoting the baseline and intervention phase, respectively. The other column is called “score” and it contains the measurements of the target behavior. An example data set with the organization of the data file is presented when opening the website. The data file can be created, for instance, with Microsoft Excel and copied and pasted as a plain text file (with a .txt extension). The example datasets available at https://osf.io/qsr42/ are the ones corresponding to the illustrations used in the current text.

On this website, the user can specify the minimal baseline phase length and minimal intervention phase length. These first two aspects are relevant for determining the admissible starting points for the randomization. Additionally, it is necessary to specify the minimum and maximum values of the score for the time series plot, in order to have a graphical representation with the appropriate *Y*-axis scale (Dart & Radley, 2018).

The next step is to specify, by clicking in the left panel of the website, the expected data pattern. This step requires previous theoretical or empirical knowledge related to the content domain. Specifically, the user selects (a) whether the aim is to increase or decrease the target behavior; (b) whether the effect is expected to be immediate or delayed; (c) whether the onset is expected to be abrupt or gradual; (d) whether an improving (linear) baseline trend is expected or not; and (e) for immediate gradual temporary effects, whether the offset of the effect is expected to be abrupt, gradual, or as an asymptote. We did not include the option to select explicitly (i.e., a priori) whether the effect is expected to be permanent or temporary because we assumed that effects are expected to last throughout the duration of the intervention phase. In contrast, we included permanent and temporary effects as possible outputs (part of an exploratory a posteriori approach) when working with immediate vs. delayed and abrupt vs. gradual effects. After the user performs the selection of the expected data pattern by clicking, the response functions are generated internally automatically. That is, the user is not required to create a separate file with the response function. Only a text file with the datasets (i.e., the previously mentioned two columns) is needed, as illustrated by the welcome screen of the website.

The output of the website is a series of plots, accompanied by the rank of the value of the test statistic for the actual point of change in phase. Specifically, for a predicted immediate and abrupt effect, a permanent effect is compared to several durations for a temporary effect. Analogously, for an immediate and gradual effect, a permanent effect is compared to several durations for a temporary effect. For a delayed, abrupt, and permanent effect, several possible latencies are compared. Equally, for a delayed, gradual, and permanent effect, several possible latencies are compared.

Finally, it is also possible to specify a user-defined response function that depicts some other kind of expected effect, apart from the previously mentioned default response functions. For making this possible, the researcher needs to load a simple text file, with only one line, containing the numbers that define the response function for the intervention phase. The line has to contain as many numbers as the maximum possible intervention phase length. These numbers need to be separated by commas. For the baseline phase, the value of zero is assumed for all measurement occasions. For instance, if a nonlinear increase is expected during the intervention phase, following a quadratic model, the values for response function would be {1, 4, 9, 16, 25, 36, 49, 64, 81, 100, 121, 144}, for a maximum of 12 intervention phase measurement occasions. This example of a response function is presented when opening the website.

In summary, the website provides two complementary pieces of information: a time series plot of the raw data (in order to visually inspect whether the actually observed data pattern resembles the expected data pattern) and a quantification (and a graphical representation) of the degree to which the expected effect could have equally occurred at different moments in time (not coinciding with the actual moment of intervention). Specifically, in relation to the latter point, smaller ranks can be understood as (or converted to) smaller *p* values and would indicate that the effect actually observed is (among) the largest possible considering all admissible intervention start points. The website also allows exploring temporary effects (when the onset is immediate and abrupt or gradual) and also different amounts of delay (when the effect is delayed).

## Discussion

### Applicability of the response function framework

Each of the illustrations provided refers to a single A-B comparison. Nonetheless, our proposal is also applicable to methodologically stronger designs, such as multiple-baseline and ABAB (withdrawal/reversal) designs, each of which includes several A-B comparisons. This is because it is possible to select at random the moment of change in phase for an A-B design (i.e., random intervention start point design; Levin et al., 2019), a multiple-baseline design (Levin et al., 2018) or an ABAB design (Onghena, 1992). In the multiple-baseline and ABAB designs, the basic effect is computed as many times as there are A-B comparisons (Horner & Odom, 2014) and the aim is to check whether the intervention effect is replicated. One way of integrating the results of several replications (within a participant in a reversal design and across participants in a multiple-baseline design) is to count whether the effect is present in at least 75% of the attempts (Cook et al., 2015; Maggin et al., 2013). Another way is by counting the proportion of effect sizes (computed across all A-B comparisons) that are equal to or more extreme than the observed value of the effect size (test statistic) if the null hypothesis is true. This is the logic of the randomization test, which is applicable to any kind of test statistic, including Pearson’s correlation coefficient, quantifying the association between the response function and the measurement of the target behavior. Thus, for multiple-baseline and ABAB designs, there would not be a different coding required for the response function, but merely a repetition of the coding for each separate A-B comparison.

The illustrated response functions can be used for alternation designs, if the focus is put on level, given that there are no phases, and it is not possible to refer to a within-phase trend. For instance, an ABABBABAAB alternating treatments design can be represented by a response function such as {0, 1, 0, 1, 1, 0, 1, 0, 0, 1} (with 0s representing Condition A, and 1s representing Condition B). In any case, other analytical alternatives exist for alternation designs (Lanovaz et al., 2019; Manolov & Onghena, 2018), even in the context of randomization tests (Levin et al., 2012; Manolov, 2019).

For changing criterion designs, specific randomization tests have been suggested (Ferron et al., 2019; Onghena et al., 2019; Tanious, 2022). In case the emphasis is, as usual, on the degree to which the data match the pre-established criteria (i.e., immediate and abrupt effects with no trend), the response functions would not be necessary, and the existing procedures would suffice.

The response functions represent a general framework that allows, for instance, including the study of immediate effects (see Manolov & Onghena, 2022) as a special case. Response functions can be defined to study both (a) different number of values per phase being compared; and (b) different latencies of effect; and (c) different focal data aspects, whose immediate or delayed appearance is analyzed. For instance, regarding point “a”, if there are five measurements per phase, the response function could be defined as {NA, NA, 0, 0, 0, 1, 1, 1, NA, NA} to focus on only three values per phase. Regarding point “b”, if a latency of two measurement occasions is expected, and all data are to be used, the response function could be defined as {0, 0, 0, 0, 0, 0, 0, 1, 1, 1}. Regarding point “c”, if the focus of the analysis is an immediate change in slope, including only four measurements per phase, the response function could be defined as {NA, 0, 0, 0, 0, 0, 1, 2, 3, NA}.

### Recommendations for applied researchers

#### When to apply the exploratory approach

As an initial option, we are echoing the usual recommendations (Edgington, 1975; Heyvaert & Onghena, 2014a, 2014b; Levin et al., 2021) for choosing the test statistic according to the available theoretical and empirical basis for the subject matter at hand. In context of the current proposal, what will be chosen according to the a priori expectations is not the test statistic itself (which would be Pearson’s correlation coefficient quantifying the relation between the measurements and the response function), but rather the response function. This would also correspond well with the importance of preregistration in relation to the data analytical plan (Cook et al., 2022; Manolov et al., 2022; Porcino et al., 2020). This is consistent with a hypothetico-deductive or a static approach to data analysis, although it may not be feasible when an exploratory or dynamic approach is followed (Johnson & Cook, 2019). Therefore, the more exploratory approach would be restricted to the operational definition of the delay or the duration of the effect. Thus, there would be a combination between expectations (regarding the data pattern) and exploration (regarding the specific temporal aspects of the effect).

Nonetheless, a different scenario should not be discarded. It is possible that the researchers have a first a priori specification, which ends up having insufficient correspondence with the actually observed data pattern. In such a case, a further exploration of alternative response functions is possible. Next cases can use the best response function of previous cases. Another scenario might be to specify multiple response functions a priori and test them all, correcting for multiple testing.

#### Is there a place for *p* values?

*P* values and null hypothesis statistical testing have been objects of controversy and criticism in the context of social and behavioral sciences (Cohen, 1990, 1994; Gigerenzer, 2004; Nickerson, 2000) and more specifically in the SCED context (Branch, 2014; Perone, 1999). Nevertheless, most of the criticism has been directed towards their incorrect use and interpretation, and not so much in relation to their intrinsic features. Specifically, if we focus on their informative value, most criticism refers to *p* values not answering the questions that researchers are asking, although it is not clear what these questions are (Lakens, 2021). Additionally, it is not clear that suggested alternatives such as effect-size measures and confidence intervals^6^ (Wilkinson et al., 1999) are free from misinterpretations and misuses (Cortina & Landis, 2011; Greenland et al., 2016). Thus, there have been suggestions (Lakens, 2021) and efforts (Wasserstein et al., 2019; Wasserstein & Lazar, 2016) to promote the correct use and interpretation of *p* values.

In the current context, we consider that the information that the *p* value provides (i.e., the probability^7^ of a result as extreme as or more extreme than the one actually observed, in case the null hypothesis of ineffective intervention is true) is useful. However, a *p* value is not sufficient when evaluating intervention effectiveness, as it has to be considered together with the effect size (here, the value of the test statistic), visual analysis (to check whether the initial chosen response function represents well the actually observed data pattern), and an assessment of social validity (Horner et al., 2005; Kazdin, 1977). Finally, it should be highlighted that the current proposal does not consist in suggesting a way of obtaining *p* values (randomization tests have been suggested several decades ago in the SCED context; Edgington, 1967, 1975), but rather on the representation of different possible data patterns when defining the test statistic in the randomization test.

#### Reporting

Researcher degrees of freedom when making data analytical decisions (Wicherts et al., 2016) are a reality, when there is no gold standard or a sound basis for specific expectations. In the context of the need for an exploratory element in the data analytical approach, concerns can arise. One of these concerns refers to overfitting, or presenting the results for a response function and a specific operational definition of the temporal component (latency for delayed effects or duration for temporary effects) that is only applicable to the data at hand. This is also related to selective reporting (Kratochwill et al., 2018; Laraway et al., 2019; Tincani & Travers, 2022) of the results that present the best fit, even though they could be only one of the instances checked. The problem would arise only if we assume that such selective reporting takes place. Our recommendation is to report, in a transparent way, the data analytical process, including the initial expectations, how they affected the a priori choice of a response function, and whether any modifications to this choice took place once the data were gathered and visually inspected. Considering the crucial importance of the justification of the data analytical approach (Tate et al., 2013; Tincani & Travers, 2022), selective reporting is made less likely, as the researchers may not have a solid basis for any specific expectations. They would need to declare the exploratory nature of the analysis in case such an approach is followed. An exploratory approach may seem too lenient, but it appears to be a better option than selecting only one response function and sticking to it regardless of whether there is any basis for this choice and whether this response function is adequate for the data at hand. Checking, and reporting, the degree to which the conclusions change according to the operative definition may be more informative.

### Limitations

Selecting a response function prior to gathering the data reflects the common requirement, in the context of randomization tests, to choose the test statistic before data collection, according to the expected effect (Edgington, 1975; Heyvaert & Onghena, 2014a, 2014b; Levin et al., 2017). This entails following a deductive approach based on previous research. Thus, deciding whether the response function should include baseline trends depends on whether spontaneous improvement prior to the intervention is likely on the basis of theory or frequent in empirical studies (e.g., in rehabilitation; Krasny-Pacini & Evans, 2018). Similarly, deciding whether the response function should allow for a delayed effect may be related, for instance, to existing evidence of extinction bursts (Katz & Lattal, 2021; Shahan, 2022). Finally, whether the effect is expected to be abrupt or gradual (e.g., when studying academic performance; Maggin et al., 2018) depends on the kind of intervention and target behavior. Thus, a potential limitation of the proposal of using response functions is the possibility that there is not enough knowledge accumulated to guide the a priori choice. In that sense, the response functions would not be applicable in exploratory research or for formative data analysis (Johnson & Cook, 2019). Similarly, the application of the randomization test logic to response-guided experimentation may require adaptations, such as deciding when to change the condition once stability has been achieved (see Edgington, 1975). In any case, if a researcher is unwilling to determine the phase lengths before gathering the data or to wait for a random selecting of the moment of change in phase once stability has been obtained, the current proposal cannot be used.

The main assumption underlying the use of the response functions is that they reflect well the actual data pattern. If this is not the case, the value of Pearson’s correlation coefficient would be smaller and the *p* value associated with it would be larger. Thus, the result of the randomization test would correctly indicate that the initial expectations about the type of data pattern is not met. This is not necessarily a limitation. The mismatch between what is expected and what is actually obtained could lead to post hoc modifications in the initial data analytical plan, as would be the case regardless of whether response functions are used or an alternative data analytical technique is employed (e.g., if the plan is to use the between-case standardized mean difference by Hedges et al., 2012, 2013, but trends are observed in the data).

Any use of a randomization test requires not only the presence of randomization in the design before gathering the data but also having at least 20 possible randomizations for enabling a *p* value as small as 0.05. For a multiple-baseline design and following the Koehler–Levin procedure this can be achieved with as few as three participants and two possible (nonoverlapping) intervention start points per participant (a total of 3 !  × 2^3^ = 6 × 8 = 48 randomizations), whereas following the Wampold–Worsham procedure this is achieved by selecting one of the 4 !  = 24 possible orders of four participants with fixed intervention start points (Levin et al., 2018). For a reversal design with *I* = 4 phases (Onghena, 1992), it is possible to obtain 20 randomizations with a series length of *n* = 15 measurements and a minimal phase length of *k* = 3 measurements, as derived from the expression $$\left(\begin{array}{c}n-I\times k+\left(I-1\right)\\ {}\left(I-1\right)\end{array}\right)=\left(\begin{array}{c}15-4\times 3+3\\ {}3\end{array}\right)$$. For an alternating treatments design with block randomization (Onghena & Edgington, 2005), five blocks of two conditions (i.e., *n* = 10) are needed to achieve 2^5^ = 32 possible randomizations.

### Future research

The current text presents an approach to defining a test statistic in randomization tests that takes into account the predicted response function. The rationale for this approach is discussed and it is illustrated with real data, apart from implementing it in user-friendly software. However, we did not carry out a simulation study for studying the statistical power of the randomization test for different response functions, different definitions of the temporal aspect of delayed and temporary effects, and different data patterns (and also for different number of participants and number of measurements). Furthermore, it is possible to use different test statistics, apart from Pearson’s correlation coefficient: for instance, the sum of squared or absolute deviations between the measurements and the response function. We do consider that such a study would be a necessary next research step. A different possible test for the proposal would be its application prospectively in an empirical study. Before such tests with simulated and real data are performed, the current text should be considered an initial step in the definition of a general framework for test statistics for randomization tests.

## Footnotes

1 Actually, for some SCEDs, there are several possible randomization schemes: see Levin et al. (2018) for multiple-baseline designs, and Onghena and Edgington (2005) for alternation designs.

2 It is not even assumed that the data are independent or interchangeable because the data are not permuted, as the data sequence is maintained and what is randomized (in the design, before gathering the data, and equally when carrying out the randomization test) are the labels representing the different conditions. Whatever autocorrelation may be present in the data, it is the same under all randomizations because the data are not permuted or randomized.

3 The term “tier” is used to refer generically to participants, behaviors, or settings, in order not to have to list all three every time that we refer to multiple baseline designs.

4 For instance, if for a given tier including 15 measurements the intervention is decided at random to start either at measurement occasion 6 or 7, there are two possible randomizations before data collection and two possible ways to divide the data once gathered, before computing the test statistic for each data division: (a) having five baseline measurements and ten intervention phase measurements; and (b) having six baseline measurements and nine intervention phase measurements. For a multiple-baseline design, the total number of data divisions (randomizations) entails taking into account the number of possible intervention start points per tier and the number of tiers (see Levin et al., 2018).

5 For instance, when discussing immediate effects, the focus is usually put on the last three baseline phase measurements and the first three intervention phase measurements (Horner & Kratochwill, 2012), although some authors also suggest using five measurements (e.g., Barton et al., 2019; Wolfe et al., 2019). Actually, it has been acknowledged that the value of three is an arbitrary choice (Kratochwill et al., 2013). Logically, there is no sound reason why two or four measurements per phase should not be considered as well.

6 Randomization-based confidence intervals can be obtained by inverting the randomization test (Michiels et al., 2017).

7 Or equivalently, the proportion of possible results (under the randomization scheme) that are as extreme as or more extreme than the actually obtained result, in case the null hypothesis is true.
